# Delta-like Ligand 3–Directed ^225^Ac Radioimmunotherapy in Neuroendocrine Lung and Prostate Cancer Models

**DOI:** 10.2967/jnumed.125.271302

**Published:** 2026-06

**Authors:** Tran T. Hoang, David Bauer, Aidan Ingham, Ileana C. Miranda, Lukas M. Carter, Alexa Michel, Roberto De Gregorio, Juliana Welk, Hong Zhong, John T. Poirier, Michael J. Morris, Lisa Bodei, Charles M. Rudin, Salomon Tendler, Jason S. Lewis

**Affiliations:** 1 Department of Pharmacology, Weill Cornell Graduate School of Medical Sciences, New York, New York;; 2 Program in Molecular Pharmacology, Memorial Sloan Kettering Cancer Center, New York, New York;; 3 Laboratory of Comparative Pathology, Memorial Sloan Kettering Cancer Center, Weill Cornell Medicine, and The Rockefeller University, New York, New York;; 4 Department of Medical Physics, Memorial Sloan Kettering Cancer Center, New York, New York;; 5 Department of Medicine, Memorial Sloan Kettering Cancer Center, New York, New York;; 6 Perlmutter Cancer Center, New York University Langone Health, New York, New York; and; 7 Department of Radiology, Memorial Sloan Kettering Cancer Center, New York, New York

**Keywords:** neuroendocrine cancer, radionuclide therapy, ^225^Ac, DLL3, radioimmunotherapy

## Abstract

This work evaluated the in vivo performance of an anti–delta-like ligand 3 (DLL3) monoclonal antibody, TDI-Y-010, covalently linked to the MACROPA-derived chelator (mcp) for ^225^Ac radioimmunotherapy in 2 DLL3-positive neuroendocrine cancer models of the lung and prostate. **Methods:** Ex vivo biodistribution studies were conducted to evaluate the uptake of [^225^Ac]Ac-mcp-TDI-Y-010 and determine appropriate therapeutic dosing. On the basis of dosimetry data, 3 doses of [^225^Ac]Ac-mcp-TDI-Y-010 (9.25, 18.5, and 37.0 kBq) were evaluated in female nude mice bearing Lu149 small cell lung cancer patient-derived xenografts. An additional therapeutic efficacy study was conducted in male nude mice bearing H660 neuroendocrine prostate cancer xenografts, with administered doses of 4.63, 9.25, and 18.5 kBq. **Results:** In the Lu149 model, the median survival of the [^225^Ac]Ac-mcp-TDI-Y-010 treatment groups was significantly longer than that of the saline treatment cohorts (*P* < 0.0001 and *P* = 0.0002, respectively). In the neuroendocrine prostate cancer model, median survival was significantly longer for mice in the [^225^Ac]Ac-mcp-TDI-Y-010 groups than in those treated with [^225^Ac]Ac-mcp-IgG4 (median survival, 37 d; *P* = 0.002, 0.0001, and 0.0006 for the 4.63-, 9.25-, and 18.5-kBq [^225^Ac]Ac-mcp-TDI-Y-010 groups, respectively). Hematologic toxicity was transient in both models and comparable across all cohorts. Histopathologic assessment of background organs demonstrated mild to moderate kidney and ovary toxicity in the SC16 group compared with the highest-dose TDI-Y-010 cohort (37.0 kBq). **Conclusion:** [^225^Ac]Ac-mcp-TDI-Y-010 exhibited excellent antitumor efficacy with mild and transient hematologic toxicity, supporting its potential as a radioimmunotherapeutic agent for patients with DLL3-expressing neuroendocrine cancers.

## Visual Abstract

**Figure:**
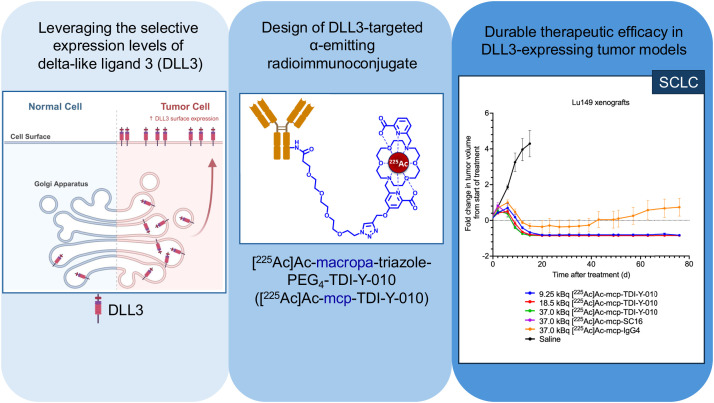

Delta-like ligand 3 (DLL3) is a Notch inhibitory ligand normally expressed at low levels in healthy adult neuronal tissues but overexpressed on the cell surface of certain tumors (1–3). DLL3 expression has been confirmed in high-grade neuroendocrine cancers, such as small cell lung cancer (SCLC), neuroendocrine prostate cancer (NEPC) (4), and other rare cancer subtypes with neuroendocrine features (5). Leveraging the tumor-selective expression of DLL3 presents an opportunity to improve outcomes in metastatic neuroendocrine cancers, which are not primarily treated with external beam radiotherapy because of their high metastatic burden. Several clinical DLL3-targeted therapies have been explored in patients with SCLC or NEPC, including antibody–drug conjugates (1,6), chimeric antigen receptor T cells, and T-cell engagers. One such agent, tarlatamab, recently received approval from the Food and Drug Administration for patients with extensive-stage SCLC (7,8) and is currently under investigation for the treatment of NEPC (9,10).

SC16, a first-generation anti-DLL3 monoclonal antibody, was initially developed for use as an antibody–drug conjugate, rovalpituzumab tesirine, but was ultimately discontinued because of dose-limiting toxicity in multiple phase 3 clinical trials (11–13). We previously repurposed SC16 as a radioimmunotherapeutic labeled with the β-emitting radionuclide ^177^Lu. The construct, [^177^Lu]Lu-CHX-A"-DTPA-SC16, showed durable antitumor responses with transient hematologic toxicity in DLL3-expressing SCLC and NEPC tumor models (14,15). In parallel, we developed a next-generation antibody optimized for radiotheranostic applications, TDI-Y-010, which binds to human DLL3 with high affinity (*K*_d_ = 5.0 nM) and exhibits superior therapeutic efficacy when radiolabeled with ^177^Lu compared with the SC16-based analog (16).

Compared with β-emitting radionuclides such as ^177^Lu, α-emitting radionuclides possess significantly higher linear energy transfer (∼80 keV/µm vs. 0.2 keV/µm), greater relative biologic effectiveness, and shorter depths of tissue penetration (40–100 µm vs. 50–12,000 µm) (3–7). This limited path length can reduce off-target toxicity, including myelosuppression, while promoting selective tumor cell killing and sparing near healthy tissues (17–19). The α-emitter most frequently used for radiopharmaceutical therapy is ^225^Ac, which has a half-life of 9.92 d and a potent decay chain yielding 4 net α-particles and 2 β-particles. These properties make ^225^Ac particularly well suited for targeted α-particle therapy when paired with vectors possessing complementary biologic half-lives (17,20–22).

The most commonly used chelator for ^225^Ac conjugation is DOTA (23). However, DOTA-based labeling is limited by the need for high chelator-to-antibody ratios and elevated reaction temperatures (80–95 °C). Although lower-temperature radiolabeling methods have been explored, they typically require longer reaction times and additional purification steps (24–29). These conditions are suboptimal for full-length monoclonal antibodies, as radiolabeling can adversely affect tertiary structure and antigen-binding affinity. Consistent with these limitations, prior work demonstrated that DOTA-based radiolabeling of SC16 with ^225^Ac resulted in low yields, poor therapeutic indices, and suboptimal in vivo efficacy (30). In contrast, the MACROPA chelator (H_2_MACROPA; mcp) exhibits improved labeling kinetics and in vivo stability for ^225^Ac, with a previous study showing high radiochemical conversions within 5 min at room temperature and excellent serum stability after 7 d (31).

The primary objective of this study was to develop and preclinically validate an anti-DLL3 radioimmunotherapeutic using ^225^Ac and the mcp chelator to determine whether this platform provides efficient radiolabeling, potent antitumor efficacy, and minimal toxicity. Rather than relying on conventional chelation strategies, this work highlights a rational workflow integrating chelator–linker selection with an antibody specifically engineered for radiotheranostic applications, targeting DLL3-expressing neuroendocrine cancers of the lungs and prostate.

## MATERIALS AND METHODS

All materials and methods used in this study are provided in the supplemental materials, available at http://jnm.snmjournals.org (32–35).

## RESULTS

### Characterization of [^225^Ac]Ac-mcp-TDI-Y-010

**FIGURE 1.**
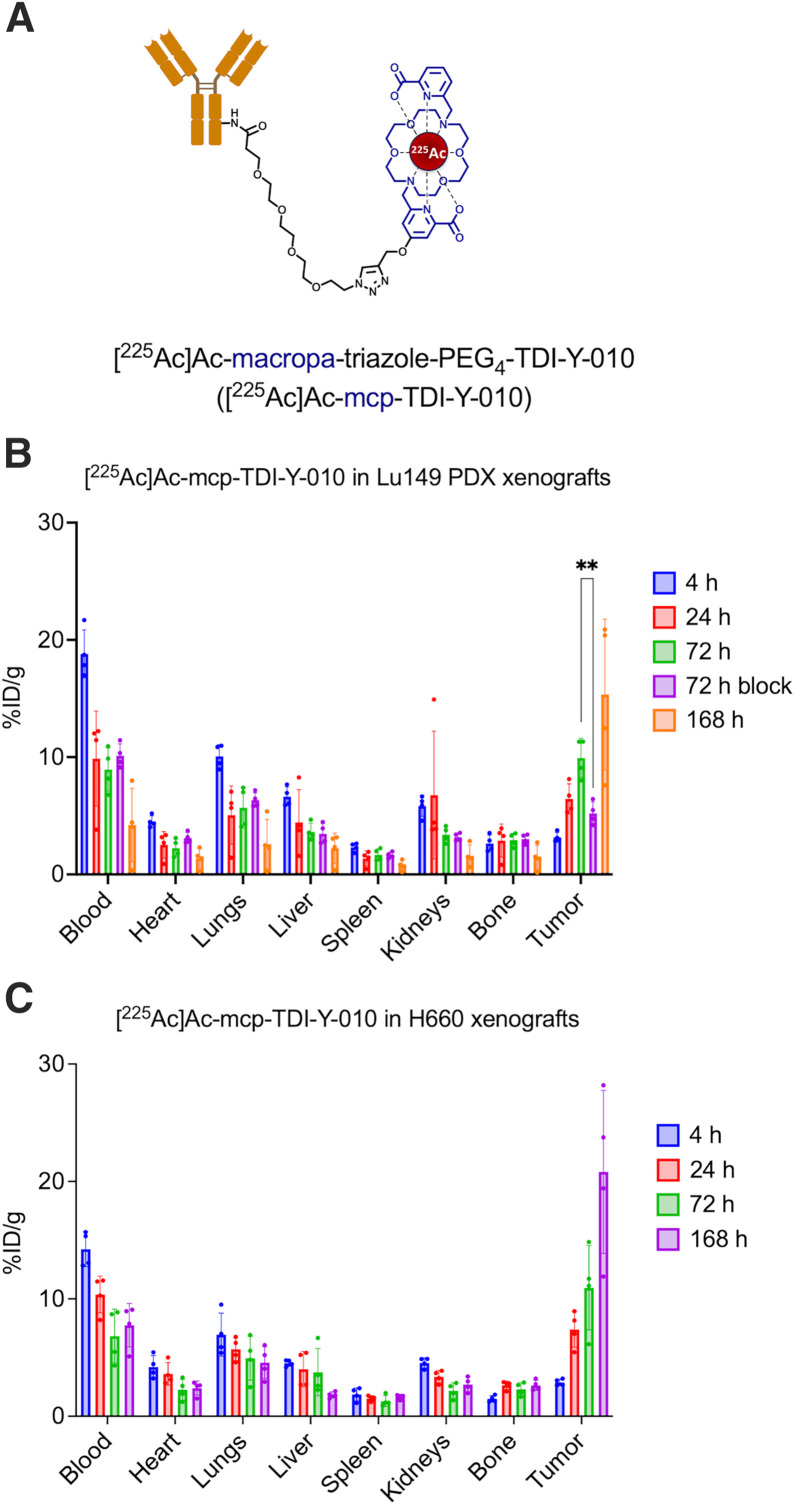
(A) Chemical structure of [^225^Ac]Ac-mcp-TDI-Y-010. Ex vivo biodistribution of select organs after administration of [^225^Ac]Ac-mcp-TDI-Y-010 (37.0 kBq; 60 µg in 110 µL of phosphate-buffered saline/HEPES) in Lu149 xenografts (*n* = 4 per time point) (B) and H660 xenografts (C) (*n* = 3 or 4 per time point). Tumor-to-kidney ratios from uptake of [^225^Ac]Ac-mcp-TDI-Y-010 in female mice bearing Lu149 xenografts (D) and male nude mice bearing H660 subcutaneous xenografts (E) (*n* = 3 or 4 per time point). **P = 0.0026.

TDI-Y-010 was nonspecifically conjugated to mcp-PEG_4_-TFP and subsequently radiolabeled with ^225^Ac to synthesize [^225^Ac]Ac-mcp-TDI-Y-010 (Fig. 1A). Matrix-assisted laser desorption/ionization time-of-flight mass spectrometry analysis showed that the average numbers of mcp chelators per TDI-Y-010, SC16, and IgG4 antibodies were 1.79 ± 0.08, 1.66 ± 0.11, and 1.83 ± 0.22, respectively (Supplemental Figs. 1A–1C). Biolayer interferometry confirmed the binding of TDI-Y-010 and TDI-Y-010-mcp to human DLL3 with subnanomolar affinities (Supplemental Fig. 2). [^225^Ac]Ac-mcp-TDI-Y-010 exhibited high radiochemical conversions when radiolabeled with ^225^Ac (>98%) and was stable when incubated in human serum at 37 °C for up to 7 d (Supplemental Figs. 3 and 4).

### Tumor Uptake in NEPC and SCLC Models

In the Lu149 SCLC patient-derived xenograft model, the administration of [^225^Ac]Ac-mcp-TDI-Y-010 (60 µg of TDI-Y-010; 37.0 kBq of ^225^Ac) resulted in progressive tumor-specific concentration of radioimmunoconjugates. Coadministration of a 7-fold excess (420 µg) of unconjugated TDI-Y-010 significantly reduced tumor uptake, confirming the construct’s specificity (*P* = 0.0026) (Fig. 1B). Tumoral uptake of [^225^Ac]Ac-mcp-TDI-Y-010 increased over time, reaching 15.3 ± 6.4 %ID/g at 168 h postinjection. Among nontumor tissues, the highest uptake was observed in the blood (4.2 ± 3.1 %ID/g at 168 h postinjection). Kidney uptake remained low (1.6 ± 0.9 %ID/g at 168 h postinjection), and all other organs exhibited minimal uptake of [^225^Ac]Ac-mcp-TDI-Y-010 (<5 %ID/g) at 168 h postinjection (Supplemental Fig. 5).

Similar results were observed in the NEPC model, as tumor uptake of [^225^Ac]Ac-mcp-TDI-Y-010 increased over time, reaching 20.8 ± 6.9 %ID/g at 168 h postinjection. Among non–tumor-bearing organs, the highest uptake of [^225^Ac]Ac-mcp-TDI-Y-010 was observed in blood (7.8 ± 1.8 %ID/g at 168 h postinjection). Kidney uptake also decreased over time (4.5 ± 0.4 %ID/g at 4 h postinjection; 2.7 ± 0.6 %ID/g at 168 h postinjection) (Fig. 1C; Supplemental Fig. 6).

### Red Bone Marrow as the Predominant Dose-Limiting Organ

Full-course biodistribution studies and dosimetry estimates identified hematologic toxicity as the most likely dose-limiting toxicity, on the basis of established normal-tissue absorbed-dose tolerances for radiopharmaceutical therapy (36). The dose coefficients for tumor tissue were 4.78 and 3.65 Gy-eq/kBq in the H660 and Lu149 models, respectively. Dose coefficients for normal tissues and therapeutic indices (i.e., tumor-to-normal tissue absorbed dose ratios) are provided in Table 1 and Supplemental Figure 7. All dose coefficients incorporated a relative biologic effectiveness weighting factor of 5 for deterministic effects of α-radiation.

**TABLE 1.** Dosimetry Values of [^225^Ac]Ac-mcp-TDI-Y-010 (37.0-kBq Dose) in Lu149 and H660 Xenografts

	Lu149 xenografts		H660 xenografts	
Tissue	Absorbed dose coefficient (Gy-eq/kBq)	Therapeutic index	Absorbed dose coefficient (Gy-eq/kBq)	Therapeutic index
Kidneys	0.627	5.8	0.759	6.3
Liver	0.784	4.7	0.678	7.1
Lungs	1.00	3.7	1.33	3.6
Red marrow	0.603	6.0	0.803	5.9
Tumor	3.65	—	4.78	—

### Dose-Dependent Tumoral Responses in the Lu149 SCLC Model

**FIGURE 2.**
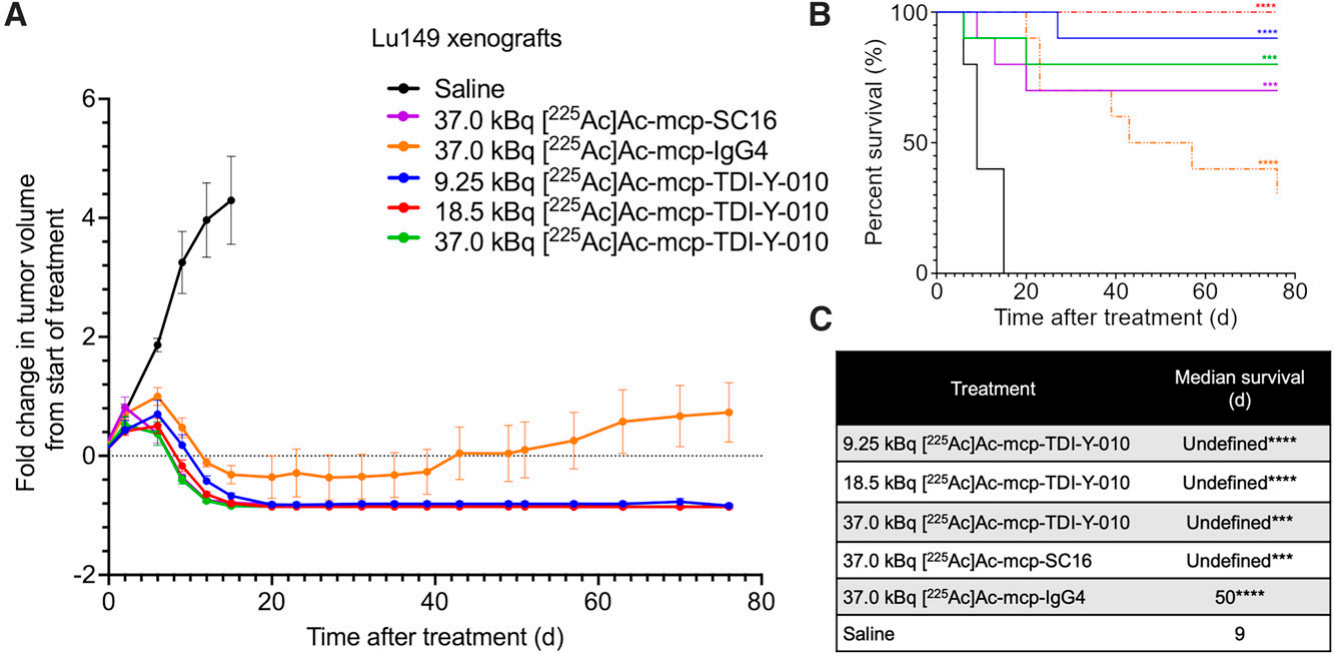
(A) Average fold change in tumor volumes after treatment initiation in female mice bearing Lu149 xenografts. Percent survival (B) and median survival (C) of female mice bearing Lu149 xenografts. Log-rank tests were performed comparing each treatment to saline cohort (*n* = 9 or 10 per cohort). Error bars represent SD. Undefined = endpoint not reached. ***P = 0.0002 for 37.0-kBq treatment group; ****P < 0.0001 for 9.25- and 18.5-kBq treatment groups.

A dose-dependent antitumor response was observed in the Lu149 SCLC model. At the end of the study (day 76), complete responses were achieved in 9 of 10 mice in the 9.25-kBq treatment cohort, all 9 mice in the 18.5-kBq cohort, and 8 of 10 mice in the 37.0-kBq cohort (Fig. 2A). The median overall survival was not reached for any group receiving [^225^Ac]Ac-mcp-TDI-Y-010, with highly significant survival benefits observed across all dose levels (*P* < 0.0001 for the 9.25- and 18.5-kBq cohorts, *P* = 0.0002 for the 37.0-kBq cohort) (Figs. 2B and 2C).

In the group receiving the lowest dose (9.25 kBq) of [^225^Ac]Ac-mcp-TDI-Y-010, 1 mouse (462) was euthanized on day 23 because of abdominal distention of undetermined etiology (Supplemental Fig. 8A). In the group treated with the highest dose (37.0 kBq), 1 mouse (431) experienced weight loss of 20% or greater on day 6, and 1 mouse (899) developed petechiae on day 16, both of whom met endpoints and warranted euthanasia (Supplemental Fig. 9C).

In the treatment group receiving 37.0 kBq of [^225^Ac]Ac-mcp-SC16, 7 of 10 mice had complete responses. Two mice (435 and 841) exhibited weight loss of more than 20% early in the study (days 6 and 9, respectively), warranting euthanasia (Supplemental Fig. 8D). One additional mouse (879) was euthanized because of generalized fragility on day 16.

In the treatment cohort receiving 37.0 kBq of [^225^Ac]Ac-mcp-IgG4, 1 mouse (893) experienced weight loss of 20% or more on day 6 (Supplemental Fig. 8E). Two mice (829 and 894) were euthanized because of frailty (days 20 and 57, respectively), and 4 mice reached the predefined tumor volume endpoint (>2000 mm^3^). Four mice (831, 892, 891, and 434) remained alive at the end of the study (day 76) (Supplemental Fig. 9E).

### Serum Chemistry Analysis and Histologic Examination in the SCLC Model

Liver enzymes (alkaline phosphatase, aspartate transaminase, and alanine transaminase levels) for most mice were within or below the reference range after therapy termination on day 76 (*n* = 3 per cohort) (Supplemental Figs. 10A–10C).

**FIGURE 3.**
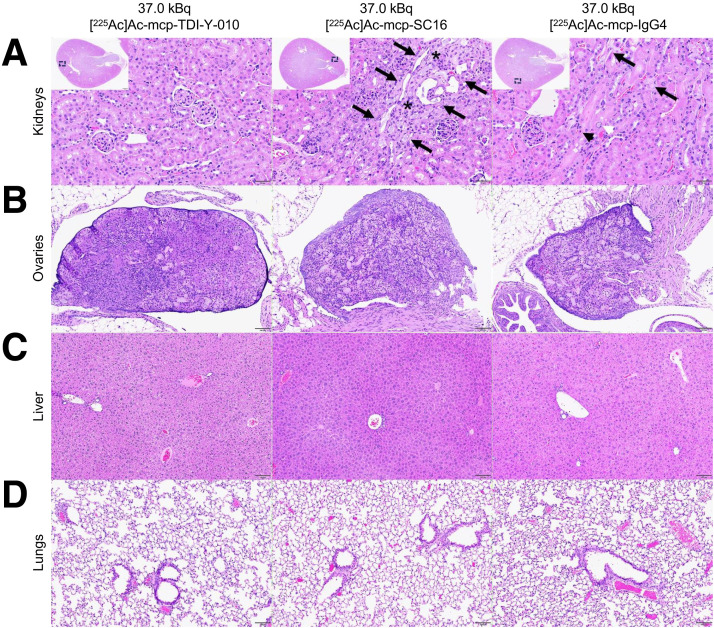
Representative histologic images of kidneys, ovaries, liver, and lungs stained with hematoxylin and eosin. (A) Kidney of mouse treated with 37.0 kBq of [^225^Ac]Ac-mcp-TDI-Y-010 is normal (scale bar: 50 µm; objective: ×20). Kidney of mouse treated with 37.0 kBq of [^225^Ac]Ac-mcp-SC16 shows area of moderate tubular damage, including tubular ectasia with occasional presence of luminal amphophilic material, epithelial cell attenuation, loss of brush borders (arrows), and minimal interstitial inflammatory infiltrates (asterisk) (scale bar: 50 µm; objective: ×20). Kidney from mouse treated with 37.0 kBq of [^225^Ac]Ac-mcp-IgG4 shows area of mild tubular damage, including tubular epithelial attenuation (arrows), single tubular epithelial cell necrosis (black arrowhead), and tubular epithelial karyomegaly (white arrowhead) (scale bar: 50 µm; objective: ×20). (B) Ovaries from all 3 groups are atrophied, consisting of decreased ovarian size with conspicuous absence of oocytes, developing follicles, or corpora lutea, with few remaining luteal, spindled, and vacuolated interstitial cells; atretic follicles; and mild lipofuscin accumulation; scale bar: 100 µm; objective: ×10. (C and D) Liver and lungs from all 3 groups are normal; scale bar: 100 µm; objective: ×10.

Histopathologic examination revealed no renal toxicity in the 37.0-kBq [^225^Ac]Ac-mcp-TDI-Y-010 cohort. In contrast, the 37.0-kBq [^225^Ac]Ac-mcp-SC16 cohort demonstrated variable degrees of subacute to chronic cortical tubulointerstitial injury, affecting a small portion of the renal tubules (≤25%), constituting the cortex or corticomedullary junction. Histopathologic findings included tubular degeneration with epithelial cell attenuation, loss of brush borders, single-cell necrosis, karyomegaly, and interstitial fibrosis. Macroscopic and microscopic alterations observed in the liver, lung, and other healthy organs were considered spontaneous background lesions commonly observed in nude mice and therefore not considered treatment-related toxicity. ^225^Ac-associated histopathologic changes were also observed in the ovaries for all 3 therapeutic cohorts, characterized by diffuse and bilateral ovarian atrophy with follicular degeneration (Fig. 3; Supplemental Fig. 11).

### High Therapeutic Efficacy with Minimal Toxicity in a NEPC Model

In the SCLC model, no significant body weight loss was observed with the lowest administered dose (9.25 kBq) of [^225^Ac]Ac-mcp-TDI-Y-010, supporting the evaluation of lower doses for antitumor efficacy in the NEPC model.

**FIGURE 4.**
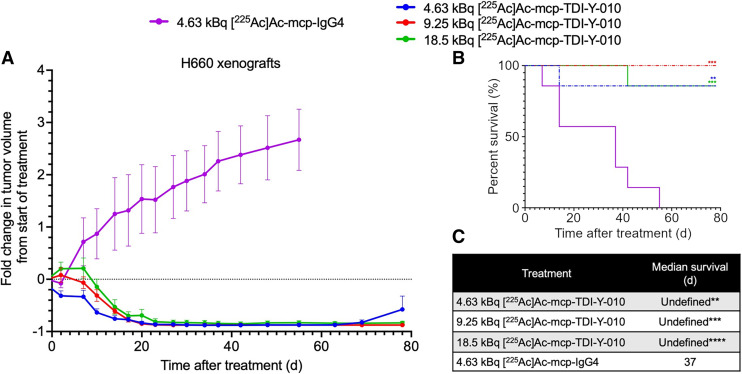
(A) Average fold change in tumor volumes after treatment in male mice bearing H660 xenografts. Percent survival (B) and median survival (C) of male mice bearing H660 xenografts after treatment. Log-rank tests were performed to compare each treatment with [^225^Ac]Ac-mcp-IgG4 cohort (*n* = 7 per cohort). Error bars represent SD. Undefined = endpoint not reached. **P = 0.002; *** P = 0.0006; **** P = 0.0001.

Of the mice treated with [^225^Ac]Ac-mcp-TDI-Y-010, complete responses were observed in 6 of 7 mice in the 4.63-kBq treatment cohort, all 7 mice in the 9.25-kBq treatment group, and 6 of 7 mice in the 18.5-kBq treatment cohort (Fig. 4A; Supplemental Fig. 12). One mouse (456) in the 4.63-kBq treatment cohort reached the tumor volume endpoint (>2000 mm^3^) on day 14, warranting euthanasia. One mouse (496) in the 18.5-kBq treatment cohort was euthanized on day 42 because of keratoacanthoma, which was deemed unrelated to treatment (Supplemental Figs. 12A and 12C).

The median OS for the [^225^Ac]Ac-mcp-IgG4 treatment cohort was 37 d. The median OS was not reached for any [^225^Ac]Ac-mcp-TDI-Y-010 treatment group and was significantly prolonged compared with the control group (*P* = 0.0006, *P* = 0.0001, and *P* = 0.002 for the 18.5-, 9.25-, and 4.63-kBq treatment groups, respectively) (Figs. 4B and 4C). No significant bone marrow toxicity or treatment-related weight loss was observed across treatment cohorts (Supplemental Fig. 13).

### Transient Hematologic Toxicity in SCLC and NEPC Models

**FIGURE 5.**
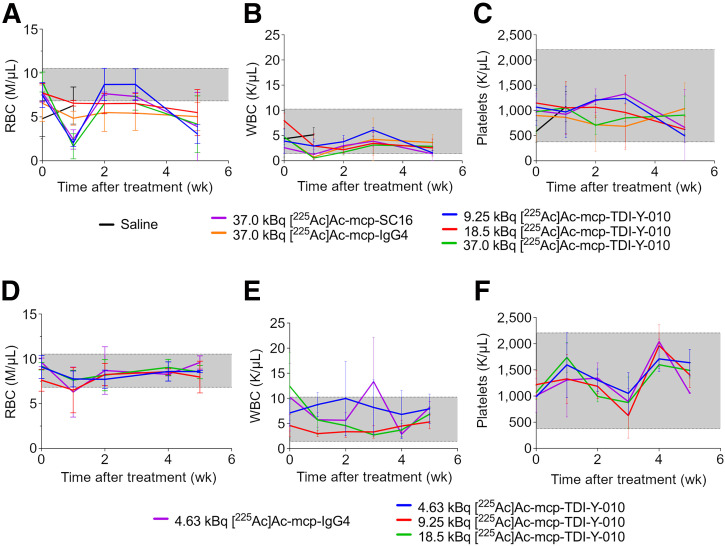
Mean red blood cell, white blood cell, and platelet counts of female nude mice bearing Lu149 xenografts (*n* = 2–4 per cohort) (A–C) and male nude mice bearing H660 xenografts (*n* = 2–4 per cohort) (D–F) after treatment. Error bars indicate SD. Shaded region represents healthy range for nude mice. Weekly blood analysis was discontinued 5 wk after study initiation in both tumor models.

In both models, red blood cell counts reached nadir 1 wk after treatment initiation in most treatment cohorts but subsequently recovered to baseline levels (Figs. 5A and 5D). White blood cell and platelet counts remained within the reference range throughout the study in the NEPC model (Figs. 5B, 5C, 5E, and 5F).

## DISCUSSION

Patients with neuroendocrine cancers have a critical unmet need for new therapeutic options, as only incremental improvements have been made in patient outcomes. Recent advances in the radiotheranostics field include the Food and Drug Administration’s approval of the peptide conjugate vipivotide tetraxetan paired with β-emitter ^177^Lu ([^177^Lu]Lu-PSMA-617) for patients with chemotherapy-naïve, prostate-specific membrane antigen–positive metastatic castration-resistant prostate cancer, highlighting progress (37).

Although β-emitters have several advantages, the high linear energy transfer and short particle path length of α-emitters may enable more effective tumor eradication, especially in chemotherapy- and radiation-resistant tumors. α-emitters have already demonstrated clinical efficacy in patients whose disease progressed during β-emitter–based radiopharmaceutical therapy (20).

[^225^Ac]Ac-mcp-TDI-Y-010 demonstrated excellent radiolabeling properties, enabling rapid progress without purification. A short tetraethylene glycol linker accelerates blood clearance and increases tumor uptake, improving tumor-to-background ratios compared with DOTA-based constructs (38). Biodistribution studies demonstrated a high tumor-to-background ratio in both tumor models. We note that administration of a therapeutically active dose (37 kBq) may influence tumor uptake at later time points as a result of treatment-induced tumor regression, potentially inflating the percentage of injected dose per gram and overestimating calculated therapeutic indices. Early time-point data, obtained before significant tumor shrinkage, remain reliable and support the targeting specificity of the DLL3-directed construct (30). Overall, our radioimmunotherapeutic agent demonstrated excellent efficacy in both SCLC and NEPC models, with most mice across all treatment groups achieving complete responses.

Hematologic toxicity occurred only at the highest administered dose (37.0 kBq) in the SCLC model, consistent with our dosimetry analysis. Tumor responses in the isotype control treated with 37.0 kBq could potentially be amplified by the enhanced permeability and retention effect (39). Nevertheless, minimal, dose-dependent hematologic toxicity was observed across treatment cohorts, with blood counts returning to baseline at 2–3 wk after treatment initiation.

Most clinical ^225^Ac radiotherapeutics use a DOTA-based chelator, such as [^225^Ac]Ac-DOTA-J591 and [^225^Ac]Ac-DOTA-617, which are under investigation in trials for treatment of metastatic castration-resistant prostate cancer (NCT03276572 and NCT04597411, respectively). An mcp-based conjugate ([^225^Ac]Ac-pelgi; Bayer) is also being evaluated clinically in metastatic castration-resistant prostate cancer (NCT06052306), with a different linker–chelator structure than ours (40). Biodistribution studies were not conducted for [^225^Ac]Ac-mcp-SC16, as SC16 was included primarily as a historical positive control rather than the investigational construct. TDI-Y-010 was prioritized for in-depth evaluation, given its optimized properties for radiopharmaceutical development and translational relevance.

Several α-particle therapy agents targeting DLL3 are in development, using DOTA chelators combined with either ^225^Ac and a fusion antibody, variable heavy chain fragment ([^225^Ac]Ac-ABD147; Abdera; NCT06736418), or a different α-emitter, ^212^Pb, with a designed ankyrin repeat protein ([^212^Pb]Pb-Radio-DARPin; Molecular Partners) (41). Each vector has distinct advantages and limitations. Full-length monoclonal antibodies, like ours, typically achieve higher tumoral uptake than do smaller molecules, critical for targets like DLL3 with low levels of surface expression (42).

A limitation of ^225^Ac radiotherapy is the potential redistribution of radioactivity resulting from the recoil energy of the ^213^Bi daughter, which can exceed the chelator’s binding threshold and release ^213^Bi from the radiopharmaceutical. This may lead to ^213^Bi accumulation in the kidney’s proximal tubules, contributing to chronic kidney toxicity (43–45). Prior studies reported increased liver uptake of ^213^Bi when chelation was suboptimal; mcp chelators improve ^213^Bi retention at room temperature, thereby enhancing efficacy and tolerability (43). Conversely, an mcp chelator has demonstrated effective in vivo stability and favorable biodistribution in preclinical studies (40). In our study, the lungs and liver received radiation, yet histopathology showed no toxicity. These findings are exploratory, given the small sample cohort sizes. Future longitudinal studies should further evaluate the kidneys as a potential dose-limiting organ for ^225^Ac and ^213^Bi. We acknowledge that the 76-d study duration was insufficient to fully assess late or cumulative hepatic and renal toxicities. Longer-term studies, including serum biochemistry, will be essential for definitively characterizing potentially delayed organ effects after treatment with [^225^Ac]Ac-mcp-TDI-Y-010.

Histopathologic analysis revealed chronic ovarian toxicity across all treatment groups. As radiosensitive organs, ovaries can undergo atrophy caused by radiation-induced irreversible injury to germ cells (46,47). Although the ovaries are not typically evaluated in radiotoxicity studies, their proximity to the kidneys may result in cross-fire irradiation from ^213^Bi daughters of ^225^Ac, leading to secondary exposure. This effect appears specific to α-emitting radioimmunotherapy and has not been observed with DLL3-directed antibody–drug conjugates, indicating a modality-dependent rather than target-dependent mechanism. Similar findings have been reported in other preclinical radioimmunotherapy studies involving mice (48–51). Ovarian off-target lesions are likely also a result of the redistribution of ^225^Ac daughters in vivo (52). Future studies should investigate other sex-dependent differences in mice after radiation exposure, as previous reports have observed differences in survival outcomes (53).

Overall, by leveraging the favorable radiolabeling properties of the MACROPA chelator, the construct demonstrated high radiochemical conversion, robust tumor uptake, and potent antitumor efficacy across 2 DLL3-expressing xenograft models. Future studies should further validate the results in other DLL3-expressing cancers, such as gastrointestinal pancreatic cancer (54) and distinct subtypes of glioma (55).

## CONCLUSION

[^225^Ac]Ac-mcp-TDI-Y-010 exhibited excellent antitumor efficacy with mild and transient hematologic toxicity, supporting its potential as a radioimmunotherapeutic agent for patients with DLL3-expressing neuroendocrine cancers.

## DISCLOSURE

This study was supported in part by National Institutes of Health (NIH) grants R35 CA232130 (Jason Lewis), R01 CA213448 (John Poirier), R35 CA263816 (Charles Rudin), U24 CA213274 (Charles Rudin), and T32 GM141949 (Tran Hoang) and NIH Cancer Center Support Grant P30 CA998748. Jason Lewis, Charles Ruin, and Memorial Sloan Kettering Cancer Center have licensed DLL3 technology to Daiichi Sankyo. Salomon Tendler, John Poirier, Charles Rudin, and Jason Lewis are named as inventors on patent applications related to anti-DLL3 antibodies, including US20240368269A1 (anti-DLL3 antibodies and uses thereof) and anti-DLL3 antibodies (patent no. 63/240,237). Jason Lewis reports research support from Clarity Pharmaceuticals and Avid Radiopharmaceuticals; has acted as an adviser to α-9 Theranostics, Clarity Pharmaceuticals, Earli, Evergreen Theragnostics, Inhibrix, Precirix, and Telix Pharmaceuticals; is a coinventor on technologies licensed to Diaprost, Elucida Oncology, Theragnostics, CheMatech, Clarity Pharmaceuticals, Daiichi Sankyo, and Samus Therapeutics; is a cofounder of pHLIP; holds equity in Summit Biomedical Imaging, Telix Pharmaceuticals, Clarity Pharmaceuticals, and Evergreen Theragnostics; and is a recipient of the TACTICAL Award from the Prostate Cancer Foundation. Charles Rudin has consulted regarding oncology drug development with AbbVie, Amgen, AstraZeneca, D2G, Daiichi Sankyo, Epizyme, Genentech/Roche, Ipsen, Jazz Pharmaceuticals, Kowa, Lilly, Merck, and Syros and serves on the scientific advisory boards of Auron, Bridge Medicines, DISCO, Earli, and Harpoon Therapeutics. Michel Morris has served as a consultant for Lantheus, Convergent Therapeutics, Z-α Therapeutics, Flare Therapeutics, Fusion Pharmaceuticals, Transtherabio, Exelixis, Amgen, Molecular Partners, Wren Laboratories, Isotopia, Actinium Pharmaceuticals, AdvanCell, Artbio, Bristol Myers Squibb, and AbbVie on drug development. Salomon Tender is the recipient of the Young Investigator Award from the Prostate Cancer Foundation and the Fellowship Award from the Druckenmiller Center for Lung Cancer Research.

## Notes

KEY POINTS **QUESTION:** Can ^225^Ac radioimmunotherapy targeting DLL3 be efficacious in mouse models of neuroendocrine cancers? **PERTINENT FINDINGS:** Durable tumor responses with transient hematologic toxicity were observed in tumor models of DLL3-expressing SCLC and NEPC using an ^225^Ac-labeled radioimmunoconjugate. **IMPLICATIONS FOR PATIENT CARE:** The findings of this study highlight the potential of [^225^Ac]Ac-mcp-TDI-Y-010 for clinical translation as a feasible treatment strategy for patients with DLL3-expressing malignancies.
